# Density-dependence in the declining population of the monarch butterfly

**DOI:** 10.1038/s41598-017-14510-w

**Published:** 2017-10-24

**Authors:** Lorenzo Marini, Myron P. Zalucki

**Affiliations:** 1 DAFNAE, University of Padova, Viale dell’Università 16, 35020 Legnaro, Padova, Italy; 2 School of Biological Sciences, The University of Queensland, St. Lucia, Queensland, 4072 Australia

## Abstract

The Eastern monarch butterfly population has significantly declined over the last two decades creating growing concerns around its conservation status. Here, we showed that the overwintering population exhibited a negative density-dependence (i.e. a negative effect on growth rate of the density in the previous year) and that, after accounting for the density effect, the population growth rate tended to decline over time. The negative time effect is probably linked to the host plant (i.e. milkweed) decline in North America. A negative density-dependence was also found in the time series of both egg density per host plant and adult density across North America suggesting the importance of a bottom-up, resource-driven regulation such as host plant limitation and/or of a top-down regulation through generalist natural enemies or diseases. The temporal stability of the density effect indicated that the negative density-dependence and the population decline are likely independent phenomena. One of the most common conclusions of previous research is that environmental stochasticity is the dominant key compounded driver of population dynamics. We showed that density dependence explained 37–50% of the total variation in growth rate in three independent datasets, indicating that several non-exclusive density-related mechanisms can be important in monarch population dynamics.

## Introduction

The monarch butterfly, *Danaus plexippus* L., is perhaps the most widely recognized of all butterflies. The status in part reflects the fascination of the public with the unique biology of the species, namely the spectacular overwintering aggregations of adults in Mexico, the annual migration in North America to breed on milkweeds and then the fall migration back to Mexico^1^. The Eastern monarch butterfly population fluctuates greatly from year to year and has shown a consistent and significant decline^2^, causing growing concerns over its conservation status^3^. The causes of the decline and even the population decline in the breeding areas have been mired in some controversy^4–6^. Most previous studies have aimed at understanding the causes of the decline in population size^5^, while no study to our knowledge has looked at the potential drivers of the inter-annual variation in population growth rate^7^. From a population dynamics perspective the two aims are clearly distinct but complementary. The first assumes non-stationarity and tries to investigate whether carrying capacity is declining due to environmental changes (e.g. host plant, pesticides, climate change) or to find other demographic factors than can bring the population close to extinction. The second approach investigates the endogenous and exogenous factors that determine the change in population growth rate from one year to the following irrespective of any general trends in abundance^8^. Surprisingly, we know little about how population growth rate has fluctuated in the last two decades during the observed population decline.

As with most invertebrate species, monarch butterfly population growth rate is affected by environmental stochasticity. Large-scale climate fluctuations can influence breeding populations by affecting individual development time, voltinism^9,10^, reproduction^11^, migration and host plant quality^12^. Moreover, extreme weather can cause mass-mortality events in the overwintering sites in Mexico^2^. On the other hand, a variety of biotic factors are known to influence both larval and adult survival. Predators^13^, parasitoids^14^ and parasites^15^, in particular, are thought to strongly limit population growth rates. Besides the processes described above, the estimated decline in the host plant (i.e. milkweed) from the early 1990’s to now has been estimated to be c. 75%^16–18^. Potentially, this decline may cause increased competition for food among larvae leading to decreases in immature survival^19,20^. While most previous studies have measured and explained vital rates independent of population size, several processes described above could influence monarch population dynamics operating in a density-dependent manner. The fact that density-dependence can operate at different stages of the life cycle suggests that studying density processes is particularly challenging for migratory species^21^. However, in the case of the monarch butterfly, the monitoring of overwintering, breeding and migrating population offers one of the few cases where the whole population can be actually observed^22^.

**Figure 1:**
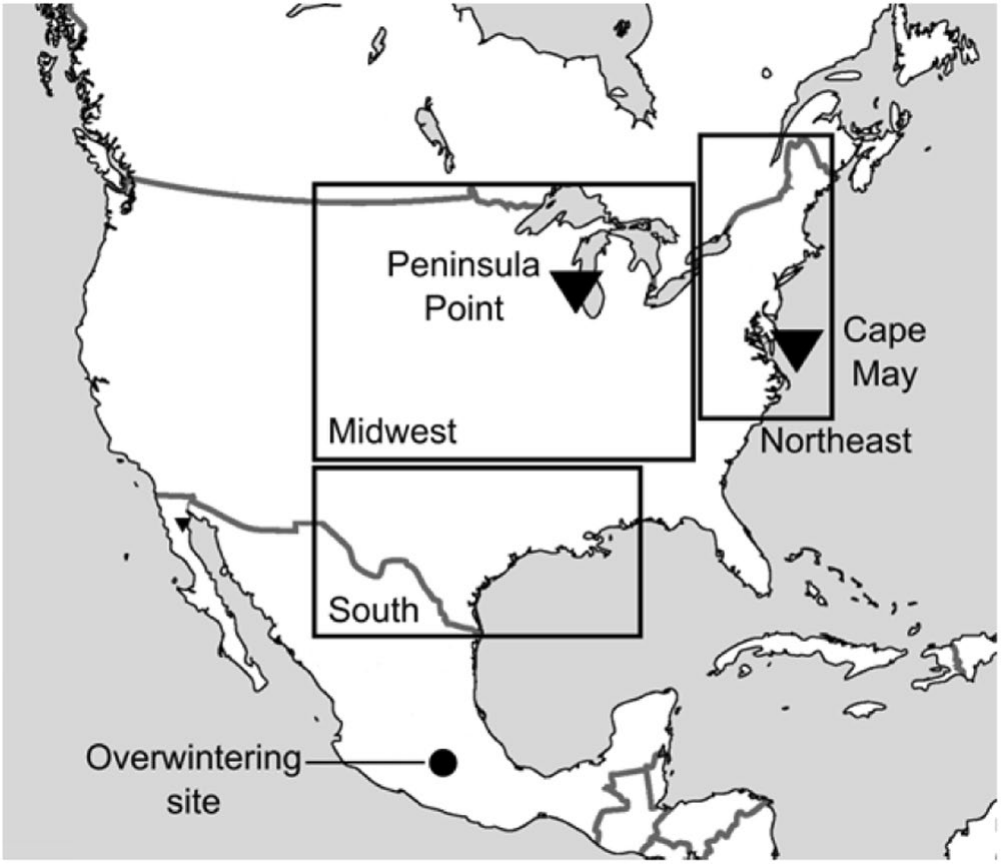
Spatial distribution of the time-series used in this study^5,24,26^. The closed dot indicates the area where the whole monarch population overwinters. The rectangles indicate the macro-regions where the butterfly breeds starting from the South in spring and moving to Midwest and Northeast in summer. The triangles indicate two major funnelling points for southern migrating monarchs. In the South we had separate time-series for spring breeding population and for fall migrating population. The map was generated using the ‘maps’ package^55^ implemented in R version 2.13.0^53^.

Although several studies have analysed the monarch population dynamics and trends using intensive monitoring data from single and multiple locations^5,7,20,23–25^, no study has investigated the inter-annual fluctuations in population growth rate. First, we tested the presence of a negative density-dependence in the population dynamics of the overwintering population in Mexico^26^, of the adult population in North America^5^, and in the available time-series of egg density per host plant across the breeding range^24^ (Fig. 1). Second, we tested whether the population growth rate, after accounting for the density effect, varied over time and whether the potential density-dependence has became more pronounced in recent years. A direct (one-year lag) density-dependence would indicate the importance of a resource-driven, bottom-up regulation such as competition for host plants or top-down regulation through predation by generalists^14^ or disease attacks^27^. A delayed density-dependence (two-year lag) would indicate the presence of a top-down regulation by specialized natural enemies that can respond numerically to monarch population fluctuations^8,28^. Our results will provide new insights on the drivers of population dynamics of this endangered population that can be used to improve the conservation efforts currently in place.

## Results

### Overwintering population in Mexico

**Figure 2:**
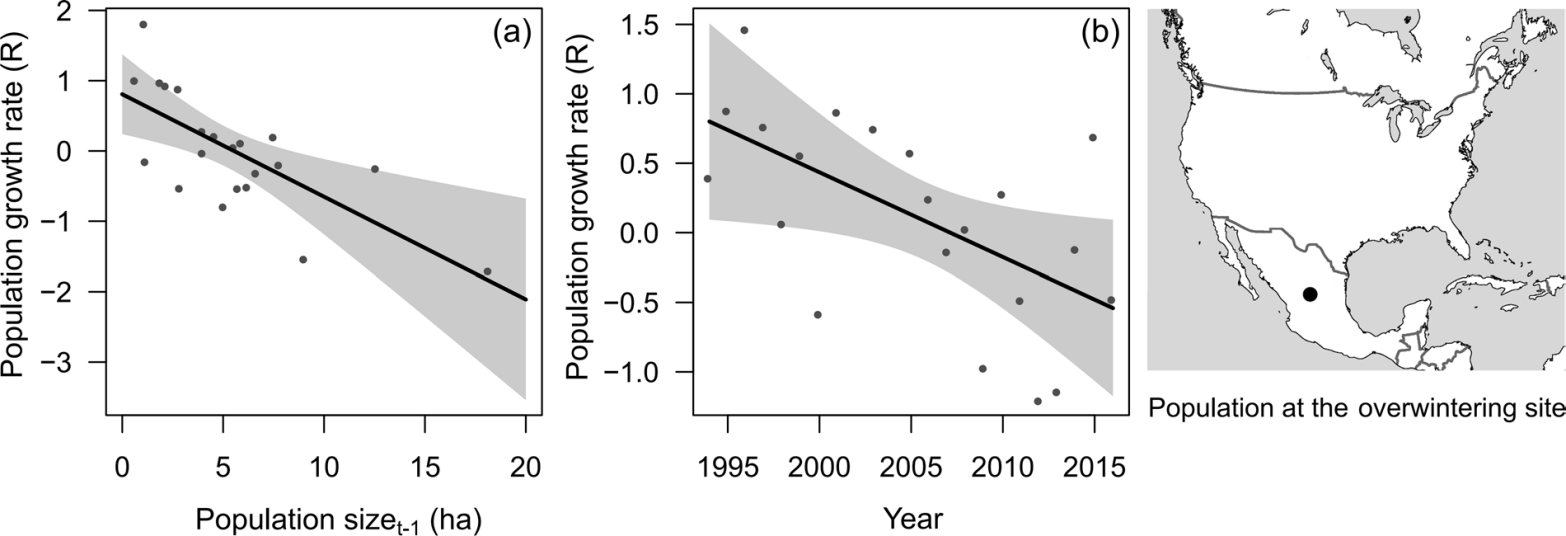
(**a**) Relationship between population growth rate and overwintering population size (*N*_*t*-*1*_) in Mexico in the previous year and (**b**) between population growth rate and time. The fitted line represents the partial model estimates from a linear model including the main effects of *Time* and *N*_*t-1*_ as explanatory variables. Dots represent the value of the variable on the x-axis and the change in response on the y-axis, holding the other variable constant. The maps were generated using the ‘maps’ package^55^ implemented in R version 2.13.0^53^.

The multi-model inference analysis indicated relatively low model selection uncertainty with two best models (ΔAICc < 2). Both models were equally supported and included both a time and density effect (Table 1). The interaction *Time* × *N*_*t-*1_ was included only in the second best model, but the low model weight (0.252) and the low log-likelihood drop indicated low support for this interaction. We found a clear negative effect of population size in Mexico in the previous year on population growth rate (Fig. 2a). After accounting for the negative density effect, we found that the population growth rate declined over time (Fig. 2b). The standardized model estimates indicated that the density effect was stronger than the year effect (Table 2). The best model including both main effects explained c. 37% of the total variation in population growth rate.

**Table 1:** Best candidate models from the multi-model procedure explaining growth rate of the overwintering population in Mexico, growth rate of the adult population in North America, and of egg density per host plant in North America. Models are ranked according to their second-order Akaike’s information criterion (AICc). Only models with ΔAICc < 7 are shown. Log-likelihood (logLik), R^2^ (or marginal pseudo-R^2^ for lme) and model weights are also reported. In all analyses, we removed two years for which the population was affected by severe winter storms in Mexico (2002 and 2004). *df* indicated the number of estimated parameters calculated as the number of fixed effect coefficients + number of variance parameters.

Rank	Fixed effects	Random effects	ΔAICc	df	logLik	R^2^	Model weight
**Overwintering population in Mexico (Model 3: linear model)**							
1^st^	*N*_*t-1*_ + *Time*	—	0	4	−17.892	0.3677	0.502
2^nd^	*N*_*t-1*_ + *Time* + *N*_*t-1*_ x *Time*	—	1.38	5	−16.833	0.4283	0.252
3^rd^	*N*_*t-1*_	—	2.40	3	−20.635	0.1789	0.151
4^th^	—	—	3.79	2	−22.705	0	0.075
5^th^	*Time*	—	6.51	3	−22.693	0.0011	0.019
**Adult population in North America (Model 4: linear mixed-effects model)**							
1^st^	*Density*_*t-1*_	*Site*	0	4	−101.39	0.5035	0.592
2^nd^	*Density*_*t-1*_ + *Time*	*Site*	1.56	5	−101.05	0.5072	0.272
3^rd^	*Density*_*t-1*_ + *Time* + *Density*_*t-1*_ x *Time*	*Site*	2.94	6	−100.60	0.5121	0.136
**Egg density per host plant in North America (Model 5: linear mixed-effects model)**							
1^st^	*Egg*_*t-1*_	*Site*	0	4	−95.599	0.4190	0.680
2^nd^	*Egg*_*t-1*_ + *Time*	*Site*	2.11	5	−95.486	0.4177	0.237
3^rd^	*Egg*_*t-1*_ + *Time* + *Egg*_*t-1*_ x *Time*	*Site*	4.20	6	−95.324	0.4165	0.083

**Table 2:** Model-averaged estimates and conditional confidence intervals (CI 95%) from the multi-model procedure explaining growth rate of the overwintering population in Mexico, growth rate of the adult population in North America, and growth rate based on egg density per host plant in North America. The explanatory variables were standardized (mean 0 and SD 1) before the analyses to make slopes comparable. In all analyses, we removed two years for which the population was affected by severe winter storms in Mexico (2002 and 2004).

	**Estimate**	**CI (95%)**
**Overwintering population in Mexico (Model 3**: **linear model)**		
*N*_*t-1*_	−0.6161	(−1.1536, −0.0786)
*Time*	−0.4644	(−0.9098, −0.0191)
*N*_*t-1*_ x *Time*	−0.2685	(−0.6905, + 0.1533)
**Adult population in North America (Model 4: linear mixed-effects model)**		
*Density*_*t-1*_	−0.7360	(−0.8909, −0.5812)
*Time*	−0.0581	(−0.2135, + 0.0973)
*Density*_*t-1*_ x *Time*	0.0765	(−0.0863, + 0.2394)
**Egg density per host plant in North America (Model 5: linear mixed-effects model)**		
*Egg*_*t-1*_	−0.9051	(−1.1611, −0.6492)
*Time*	−0.0527	(−0.3090, + 0.2037)
*Egg*_*t-1*_ x *Time*	0.0622	(−0.1613, + 0.2858)

### Adult population density in North America

**Figure 3:**
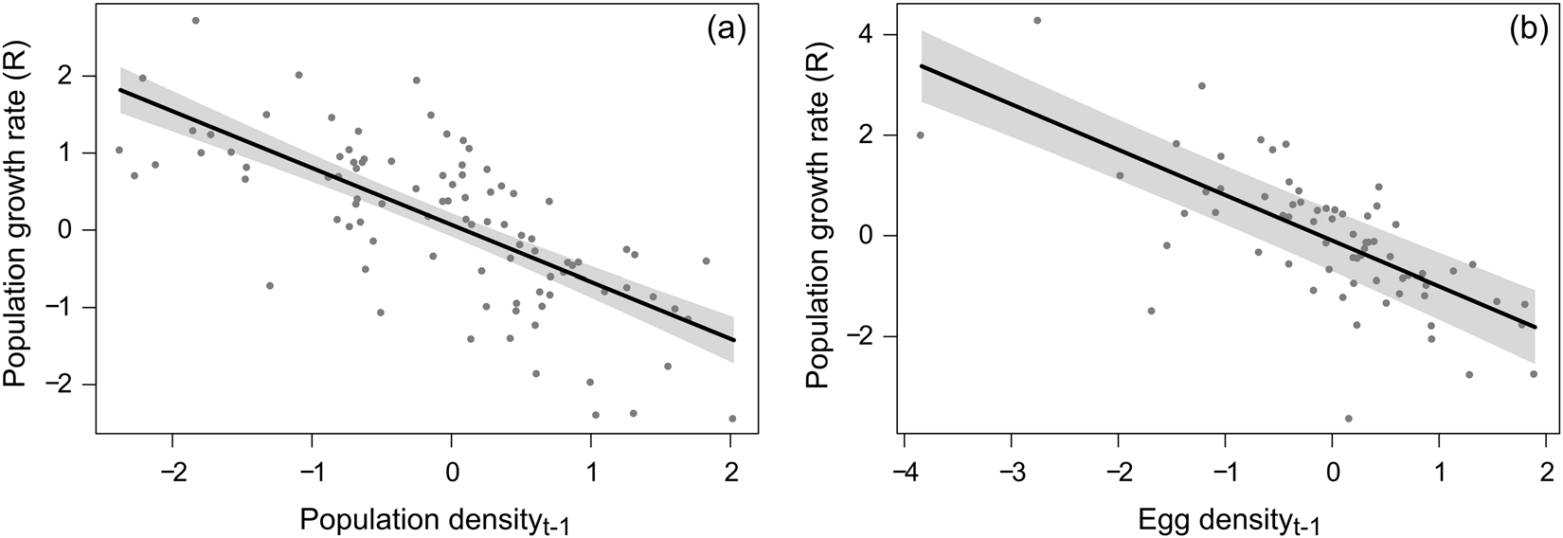
(**a**) Relationship between population growth rate (adult density) and population density (*Density*_*t-1*_) in the previous year (six time series from five sites: South, Midwest, Northeast, Cape May and Peninsula Point) and (**b**) relationship between population growth rate (egg density) and egg density per host plant (*Egg*_*t-1*_) in the previous year (five time series from three sites: South, Midwest and Northeast). The fitted line represents the model estimates from a linear mixed-effects model including the main effect of either *Density*_*t-1*_ or *Egg*_*t-1*_ and the random effect of *Site*. Both *Density*_*t-1*_ and *Egg*_*t-1*_ were ln-transformed and standardized to mean 0 and SD 1 before the analyses.

The analyses of the population density in North America indicated relatively low model selection uncertainty with two best models presenting ΔAICc < 2 (Table 1). Both models explained over half of the total variation in growth rate and both included a negative density effect in the previous year (Fig. 3a). Contrary to the overwintering population, the *Time* effect was not supported since its inclusion in the second model caused a very small log-likelihood drop. The main effect of the density in the previous year was comparable to that found in the overwintering population (Table 2). We found no support for the *Time* × *N*_*t-1*_ interaction.

### Egg density per host plant in North America

We found low model uncertainty with only one best model (model weight: 0.685) (Table 1). The multi-model inference analysis indicated only support for a negative effect of the density of eggs per stem in the previous year (Fig. 3b). The model explained over 40% of the total variation. We found no support for a *Time* effect or for the interaction between *Egg*_*t-1*_ and *Time* (Table 2).

## Discussion

The Eastern population of the monarch butterfly has strongly declined over the last two decades in the overwintering sites. Our study found a strong and consistent negative density dependence in the population dynamics of the monarch butterfly using three independent datasets. The signal was present in the overwintering population in Mexico and in the time series of both adult population density and egg density per host plant in North America. In the overwintering population, after accounting for the density-dependence effect, the population growth rate linearly declined over time. This time effect is particularly worrying as it suggests a population decline where stochastic events can assume prominence to drive the population close to extinction^22,29^. The temporal stability of the slope of the density effect (i.e. no support for a density x time interaction) indicated that the negative density-dependence and the decline of the population are likely independent phenomena. The density effect explained c. 37-50% of the total variation in population growth rate indicating that several density-related mechanisms that had been tested only locally can actually be important to explain population dynamics. Our results provide new insights on the drivers of population dynamics of this endangered population that can be used to improve both current and future conservation efforts.

Multiple non-exclusive mechanisms can be offered to explain a direct negative density-dependence in the population dynamics of the monarch butterfly. First, disease spread is expected to work in a density-dependent manner. Both field observational^30^ and experimental studies^27,31^ found that monarch infection probability by *Ophryocystis elektroscirrha*, an obligate protozoan parasite, increased significantly with increasing larval densities and aggregation of monarchs around milkweed. At high butterfly density *O*. *elektroscirrha* loads can decrease larval survival, and cause smaller adult size, resulting in shorter adult lifespans and fewer eggs laid^15^. Moreover, parasite infection can affect migration success so that infected butterflies will travel shorter distances than uninfected individuals^32,33^. A second reason could be linked to higher mortality rates by opportunistic, generalist predators and parasitoids such as tachinid flies, that have been demonstrated to be important mortality factors for the immature stages^14,28^. A third explanation is linked to host plant limitation^20^. After leaving the overwintering site, monarchs fly north and start breeding in Texas in spring. Here, observed larval and egg density are usually higher than in the summer breeding areas^27^. Experimental work testing field-realistic larval density found small density-dependent reduction in vital rates, suggesting that intraspecific competition may occur early in the breeding season in the southern portion of the breeding range^19^. Accordingly to this mechanism, we found a negative density effect in the time-series of egg density per host plant. This effect is consistent with the negative relationship previously found between local egg density and larval survival in sites with low milkweed availability^20^.

A second finding of our study is that growth rate of the overwintering population appeared to decline over time after accounting for the population size effect. The effect was not found in the time-series from North America (both adult population and egg density) probably because these data do not represent a good estimate of the whole population size^34,35^. The negative time effect is probably linked to the host plant (i.e. milkweed) decline in North America that is reducing the carrying capacity of the breeding areas. The change in the spatial distribution of the host plants after the adoption of herbicide-resistant crops resulted in a strong decline of remaining host plants in crop areas throughout much of the monarch’s summer breeding range^18,36^. Before the advent of herbicide-resistant crops the milkweed host plants were much more abundant and scattered across agricultural landscapes. Egg and first instar predation by both terrestrial (e.g. ants, spiders, mites) and flying natural enemies (e.g. predatory coccinellids) is thought to be one of the most important mortality factors in the monarch life cycle^14^. The current larger proportion of monarch population in non-crop areas where the host plant has not strongly declined might generate higher predation compared to situations with more isolated host plants scattered in crop fields^13^ (see also^37^ for the fritillary butterfly). Several oviposition experiments suggest that monarchs avoid existing conspecific eggs when seeking oviposition sites^38^. This mechanism may provide advantages to the offspring by reducing direct effects from both exploitation and interference competition and by reducing predation, as well as indirect effects from induced chemical defences of milkweed hostplants. Egg laying behaviour and fecundity is also expected to be linked to the spatial arrangement of the host plants, i.e. the current aggregated distribution of milkweed with higher inter-patch distances is expected to result in lower realized fecundity^36,39^. Beside host plant decline, the only other potential driver that has changed systematically over time is temperature due to global warming. However, temperature warming has been demonstrated to have both positive and negative effect on population dynamics^10,40,41^ and therefore cannot fully explain a linear negative trend in population growth rate. Although several initiatives have been already implemented to increase milkweed populations in non-crop habitats, the loss of the host plant in the agricultural areas might be a key driver of population decline. Hence, restoration of large milkweed populations should focus on creating a diffuse distribution of the host plant across the summer breeding areas^7,16,17^.

One of the most common conclusions of previous research on the monarch butterfly decline is that environmental stochasticity is the key compounded driver of population decline^9,12,25,40,42,43^. Large-scale climate fluctuations can affect host plant distribution, individual reproductive success, and population growth^7,40,43^. Similarly, extreme weather events have been implicated in mass-mortality events of adult butterflies in the overwintering sites^2,42^. Besides these drivers, our analysis clearly points to the importance of density-dependent processes that had been empirically tested only at the local scale^13,19,27^ or with modelling exercises^38,39,44^. Our study suggests that more effort should be placed into incorporating density effects in future population models not just between generations but also between years^7,45^. This approach will help in deciphering the drivers of change that are leading to the rapid decline of this population. Finally, the observed negative time effect on growth rate in the overwintering population is particularly worrying as it may indicate a decline in the carrying capacity in the breeding areas^22,29,46^, where mutual reinforcement among biotic and abiotic processes may drive population size to a rapid decline.

## Methods

### Dynamics of the overwintering population

The Eastern North American monarch population exhibits a migratory behaviour from the breeding areas located in northern and central United States and southern Canada to overwintering sites in Mexico. Overwintering monarchs arriving in Mexico coalesce predominately on oyamel fir forests at densities ranging from 6.9 to 60.9 million monarchs per ha^47^. By measuring the forest area occupied by the overwintering butterflies it is possible to estimate the total population size and this has been done in a consistent fashion since 1994^26^. We used the latest time-series provided by WWF (available at http://www.wwf.org.mx, access 8^th^ May 2016) (see supplementary material Table S1). As forest area used by the overwintering population is expected to be related to population size, we used this variable as a proxy for population size (*N*). Although a certain degree of methodological inconsistencies can be present, this is the sole total population size estimate available^2,22^. Then, we built a discrete model of population dynamics as the baseline for developing and comparing competing models to explain the inter-annual variation of the population growth rate. The population growth rate (dependent variable) was defined as:

$$R=ln({N}_{t}/{N}_{t-1}),$$

where *N*_*t*_ is the forest area occupied or population size in the current year while *N*_*t-1*_ is the population size in the previous year. We hypothesized that *R* would exhibit endogenous direct negative feedback (density dependence) where:

Model 1 $$R=f({N}_{t-1})+\varepsilon ,$$

where *ε* represents sampling error in the estimation of the population density plus exogenous (i.e., density independent) effects on *R*.

We also tested delayed (two-year lag) density dependent feedback with the effect of the population density two years before.

Model 2 $$R=f({N}_{t-1}+{N}_{t-2})+\varepsilon $$

As we found no support for a delayed density dependence (general linear model *R*~*N*_*t-1*_ + *N*_*t-*2_), we proceeded to test only direct density dependence in the following models. Finally, we fitted Model 1 with *Time* (year in the time series) and the interaction between *N*_*t-1*_ and *Time*:

Model 3 $$R=f({N}_{t-1}+Time+{N}_{t-1}\times Time)+\varepsilon $$

We hypothesized that the slope of the population size effect should be more negative in the later part of the time series compared to the beginning. If supported, this interaction would indicate an exacerbation of the negative feedback even if the total population size is declining. Beside population size, we did not test any exogenous variables such as climate variables because migrating species have intricate and complex relationships with climate that can vary geographically^48^ and cannot easily be described by simple weather variables^40^.

### Dynamics of the adult population in North America

To test for density-dependence we also used available population density (*Density*) data from the breeding area in North America. This data was recently compiled using the data provided by the North American Butterfly Association (NABA) that has collected citizen science data on population density over the last decades^5^. Inamine *et al*. (2016) clustered the data into three spatially distinct regions: South (south of 34.5°N and west of 79°W), the closest to the overwintering sites, Northeast (north of 34.5°N and east of 79°W), and Midwest (north of 34.5°N and west of 79°W) (Fig. 1) (see supplementary material Table S2). Northeast and Midwest included observations on the entire summer breeding season and corresponded to the core of the breeding areas. South was temporally subdivided into two seasons: Spring south (1^st^ March through 30^th^ June, corresponding to breeding individuals migrated from Mexico), and Fall south (1^st^ September to 30^th^ November, corresponding to non-breeding migrants moving south). We also added two additional time-series: Cape May and Peninsula Point. Cape May (New Jersey), is a major funnelling point for monarchs migrating south from the Northeast, while Peninsula Point (Michigan), is a funnelling point for monarchs migrating south from both eastern and midwestern Canadian populations^5^.

Similarly to the overwintering population, we first fitted Model 1 and Model 2. As we found no support for a delayed density dependence (general linear mixed model *R*~ *Density*_*t-1*_ + *Density*_*t-2*_, random = *Site*), we proceeded to test only direct density dependence in the following models. Compared to the overwintering population time series, the NABA point data did not provide an optimal estimate of the total population size, but each time-series should provide a good estimate of local density^34^. Similarly to the overwintering population, we fitted a model testing whether the potential density-dependence varied over time:

Model 4 $$R=f(Densit{y}_{t-1}+Time+Densit{y}_{t-1}\times Time)+\varepsilon $$

### Dynamics of egg density per host plant in North America

Finally, we also retrieved recently compiled data on egg density per host plant across the breeding range by extracting the information from published figures^24^. We retrieved five time series spanning from 1997 to 2014 coming from the Monarch Larva Monitoring Project (see supplementary material Table S3). We grouped the time series in three regions (Midwest, Northeast and South). The authors associated each time series to the prevalent monarch generation (supplementary material Table S3)^24^. For each time-series, we computed a growth rate based on egg density (R_EGG_) as:

$${R}_{EGG}=ln(Eg{g}_{t}/Eg{g}_{t-1}),$$

where *Egg*_*t*_ is the density per host plant in year t *and Egg*_*t-1*_ is the density per host plant in the previous year t-1.

Similarly to the adult population models, we first fitted both a direct (*Egg*_*t-1*_) and a delayed density dependence term (*Egg*_*t-2*_). As we found no support for a delayed density dependence (general linear mixed model *R*_*EGG*_~*Egg*_*t-1 *_ + *Egg*_*t-2*_, random = *Site*), we proceeded to test only direct density dependence. Then, we fitted a model testing whether the potential density-dependence varied over time:

Model 5 $${R}_{EGG}=f(Eg{g}_{t-1}+Time+Time\times Eg{g}_{t-1})+\varepsilon $$

### Statistical analyses

For overwintering population, Model 3 was fitted using general linear models. For adult population in North America, Model 4 was fitted using linear mixed-effects models including *Density*_*t-1*_ *, Time*, and *Density*_*t-1*_ x *Time* as fixed effects and site as a random factor. In Model 4, *Density*_*t-1*_ was ln-transformed to improve linearity. For egg density per host plant, Model 5 was fitted using linear mixed-effects models including *Egg*_*t-1*_ *, Time*, and *Egg*_*t-1*_ x *Time* as fixed effects and site as a random factor. *Egg*_*t-1*_ was ln-transformed to improve linearity. Before fitting all models, we standardized the explanatory variables to mean 0 and SD 1 to make slope estimates comparable. The assumptions of the models were tested by inspecting diagnostic plots of model residuals. Residuals approximated a normal distribution and exhibited homoscedasticity. Model selection was performed using an information theoretic approach to evaluate alternative competing models involving the variables included in Models 3-5^49^. Our information-theoretic approach compared the fit of all the possible candidate models nested within the full models. Models were fitted using the maximum likelihood method. The fit of each model in the set was then evaluated using second-order Akaike’s information criterion (AICc). The best fit is indicated by AICc_MIN_, the lowest value of AICc. In a set of n models each model *i* can be ranked using its difference in AICc score with the best-fitting model (ΔAICc_*i*_ = AICc_*i*_ − AICc_MIN_). A model in a set can be considered plausible if its ΔAICc is below 2. We also computed the model weight (w_i_) as the weight of evidence in favour of each model. The weights w_i_ represent the relative likelihood of a model. For each model, we first calculated its likelihood as exp(−0.5*∆AICc_i_). The weight w_i_ for a model is its likelihood divided by the sum of the likelihoods across all models. For each variable, we also provided model-averaged coefficients and intervals of confidence (CI 95%)^50,51^. The multi-model inference analyses were performed using the ‘MuMIn’ package^52^ implemented in R version 2.13.0^53^ using the functions *dredge*(), *model*.*avg*() and *confint*().

Before fitting the models described above, we retrieved published data for severe storm occurrence in Mexico during the period 1993-2016, because a major storm causing mass-mortality can strongly affect population size irrespective of any density effect. Two major storms in early 2002 and 2004 caused recorded mass-mortality^26^. Hence, two population growth rate estimates (2001-2002 and 2003-2004) were certainly affected by these events. We therefore ran all models excluding these two years. Another major winter storm occurred in the area between the 31^st^ of January and the 2^nd^ of February 2010 but no data on observed mass-mortality events are available. The only available document reports that climatic data indicates that this major storm very likely did not cause major butterfly mortality (L. Brower, L. Fink, I. Ramirez, R. Zubieta and D. Slayback, http://monarchwatch.org). Similarly between the 8^th^ and the 9^th^ of March 2016 another storm struck the overwintering sites, heavily damaging the forest but no clear published data of mass butterfly mortality is available. We therefore did not exclude data for the years 2010 and 2016 in the models presented in the main text. However, we also ran a conservative test excluding the four years potentially affected by storms. All the results remained qualitatively similar compared to those presented in the main text (see supplementary material Tables S4–6)^54^.

### Data availability

All data analysed during this study are included in this published article and its Supplementary Information files.

## Electronic supplementary material

Supplementary Material
