# Supplementary material for: Density-dependence in the declining population of the monarch butterfly
Source: Sci Rep. 2017 Oct 24;7:13957. doi: 10.1038/s41598-017-14510-w (PMC5655678; doi:10.1038/s41598-017-14510-w)
Supplement: Supplementary file 1 — Supplementary Material [file 41598_2017_14510_MOESM1_ESM.pdf]

# Density-dependence in the declining population of the monarch butterfly

Lorenzo Marini[1,*], Myron P. Zalucki[1,2]

[1]DAFNAE, University of Padova, Viale dell'Università 16, 35020 Legnaro, Padova, Italy

[2]School of Biological Sciences, The University of Queensland, St. Lucia, Queensland, Australia, 4072

*E-mail: lorenzo.marini@unipd.it, Tel.: +390498272807, orcid.org/0000-0001-7429-7685

**Table S1** Time-series for the overwintering population in Mexico (available at http://www.wwf.org.mx, access 8th May 2016). $N_t$ is the forest area in ha covered by the overwintering butterflies in year t. $R=\ln(N_t/N_{t-1})$ is population growth rate.

| ***Year*** | $N_t$ | $N_{t-1}$ | ***R*** |
|---|---|---|---|
| 1994 | 7.81 | 6.23 | 0.226028631 |
| 1995 | 12.61 | 7.81 | 0.479085186 |
| 1996 | 18.19 | 12.61 | 0.366381843 |
| 1997 | 5.77 | 18.19 | -1.148199912 |
| 1998 | 5.56 | 5.77 | -0.037073972 |
| 1999 | 9.05 | 5.56 | 0.487166649 |
| 2000 | 2.83 | 9.05 | -1.162488046 |
| 2001 | 9.35 | 2.83 | 1.195099632 |
| 2002 | 7.54 | 9.35 | -0.215154161 |
| 2003 | 11.12 | 7.54 | 0.388523107 |
| 2004 | 2.19 | 11.12 | -1.624843745 |
| 2005 | 5.92 | 2.19 | 0.994434905 |
| 2006 | 6.67 | 5.92 | 0.119283411 |
| 2007 | 4.61 | 6.67 | -0.369392003 |
| 2008 | 5.06 | 4.61 | 0.093138626 |
| 2009 | 1.92 | 5.06 | -0.969041297 |
| 2010 | 4.02 | 1.92 | 0.738956717 |
| 2011 | 2.89 | 4.02 | -0.330025401 |
| 2012 | 1.19 | 2.89 | -0.887303195 |
| 2013 | 0.67 | 1.19 | -0.574430874 |
| 2014 | 1.13 | 0.67 | 0.522695199 |
| 2015 | 4.01 | 1.13 | 1.266573609 |
| 2016 | 2.91 | 4.01 | -0.32063816 |

**Table S2** Time-series for the population density in North America. Data are from Inamine et al.[2] $Density_t$ is population density of butterflies in year t. $R$=ln($Density_t$/$Density_{t-1}$) is population growth rate.

| *Year* | *Site* | *Density_t* | *Density_t-1* | *R* | *Life cycle phase* |
|---|---|---|---|---|---|
| 2006 | South | 77.268 | 44.629 | 0.548896026 | Breeding |
| 2007 | South | 72.977 | 77.268 | -0.057135575 | Breeding |
| 2008 | South | 51.261 | 72.977 | -0.353214094 | Breeding |
| 2009 | South | 75.296 | 51.261 | 0.384496783 | Breeding |
| 2010 | South | 29.595 | 75.296 | -0.933821584 | Breeding |
| 2011 | South | 34.3 | 29.595 | 0.147539926 | Breeding |
| 2012 | South | 20.861 | 34.3 | -0.497263967 | Breeding |
| 2013 | South | 10.31 | 20.861 | -0.704767089 | Breeding |
| 2014 | South | 21.129 | 10.31 | 0.717532206 | Breeding |
| 1994 | Midwest | 226.537 | 153.365 | 0.390087586 | Breeding |
| 1995 | Midwest | 35.737 | 226.537 | -1.84672172 | Breeding |
| 1996 | Midwest | 102.151 | 35.737 | 1.050265544 | Breeding |
| 1997 | Midwest | 230.106 | 102.151 | 0.812087962 | Breeding |
| 1998 | Midwest | 104.858 | 230.106 | -0.785933018 | Breeding |
| 1999 | Midwest | 255.704 | 104.858 | 0.891413472 | Breeding |
| 2000 | Midwest | 149.817 | 255.704 | -0.534605976 | Breeding |
| 2001 | Midwest | 307.803 | 149.817 | 0.720045419 | Breeding |
| 2002 | Midwest | 166.007 | 307.803 | -0.617430012 | Breeding |
| 2003 | Midwest | 193.017 | 166.007 | 0.150748312 | Breeding |
| 2004 | Midwest | 58.672 | 193.017 | -1.190815657 | Breeding |
| 2005 | Midwest | 163.33 | 58.672 | 1.023810083 | Breeding |
| 2006 | Midwest | 338.107 | 163.33 | 0.727589719 | Breeding |
| 2007 | Midwest | 266.017 | 338.107 | -0.239802197 | Breeding |
| 2008 | Midwest | 170.119 | 266.017 | -0.447062024 | Breeding |
| 2009 | Midwest | 185.16 | 170.119 | 0.084722124 | Breeding |
| 2010 | Midwest | 306.761 | 185.16 | 0.504848627 | Breeding |
| 2011 | Midwest | 140.353 | 306.761 | -0.781908265 | Breeding |
| 2012 | Midwest | 169.584 | 140.353 | 0.189187702 | Breeding |
| 2013 | Midwest | 41.939 | 169.584 | -1.397132198 | Breeding |
| 2014 | Midwest | 99.009 | 41.939 | 0.858994573 | Breeding |
| 1994 | Northeast | 59.704 | 39.425 | 0.414998887 | Breeding |
| 1995 | Northeast | 43.021 | 59.704 | -0.327710651 | Breeding |
| 1996 | Northeast | 37.713 | 43.021 | -0.131683506 | Breeding |
| 1997 | Northeast | 108.253 | 37.713 | 1.054466218 | Breeding |
| 1998 | Northeast | 40.951 | 108.253 | -0.97209485 | Breeding |
| 1999 | Northeast | 104.118 | 40.951 | 0.933148641 | Breeding |
| 2000 | Northeast | 80.296 | 104.118 | -0.259805065 | Breeding |
| 2001 | Northeast | 90.546 | 80.296 | 0.120138202 | Breeding |
| 2002 | Northeast | 21.381 | 90.546 | -1.443355332 | Breeding |
| 2003 | Northeast | 41.897 | 21.381 | 0.672711548 | Breeding |
| 2004 | Northeast | 16.049 | 41.897 | -0.959567683 | Breeding |
| 2005 | Northeast | 58.997 | 16.049 | 1.301840053 | Breeding |
| 2006 | Northeast | 265.467 | 58.997 | 1.504003944 | Breeding |
| 2007 | Northeast | 179.67 | 265.467 | -0.390368704 | Breeding |
| 2008 | Northeast | 132.027 | 179.67 | -0.308115388 | Breeding |
| 2009 | Northeast | 88.072 | 132.027 | -0.404851785 | Breeding |
| 2010 | Northeast | 95.789 | 88.072 | 0.083993194 | Breeding |
| 2011 | Northeast | 80.143 | 95.789 | -0.178335317 | Breeding |
| 2012 | Northeast | 178.336 | 80.143 | 0.799856872 | Breeding |
| 2013 | Northeast | 16.801 | 178.336 | -2.362231003 | Breeding |
| 2014 | Northeast | 46.367 | 16.801 | 1.015149591 | Breeding |
| 1994 | Cape May | 839.8 | 544.6 | 0.433112188 | Migrating |
| 1995 | Cape May | 248.5 | 839.8 | -1.217720923 | Migrating |
| 1996 | Cape May | 503.6 | 248.5 | 0.706339457 | Migrating |
| 1997 | Cape May | 919.6 | 503.6 | 0.602156491 | Migrating |
| 1998 | Cape May | 403.1 | 919.6 | -0.824754123 | Migrating |
| 1999 | Cape May | 2849.2 | 403.1 | 1.955608862 | Migrating |
| 2000 | Cape May | 250.7 | 2849.2 | -2.430536527 | Migrating |
| 2001 | Cape May | 658.4 | 250.7 | 0.965555644 | Migrating |

| | | | | | |
|---|---|---|---|---|---|
| 2002 | Cape May | 276.8 | 658.4 | -0.866517426 | Migrating |
| 2003 | Cape May | 392.3 | 276.8 | 0.348731629 | Migrating |
| 2004 | Cape May | 74 | 392.3 | -1.66796176 | Migrating |
| 2005 | Cape May | 538.2 | 74 | 1.984165145 | Migrating |
| 2006 | Cape May | 1743.4 | 538.2 | 1.17536227 | Migrating |
| 2007 | Cape May | 746 | 1743.4 | -0.848866908 | Migrating |
| 2008 | Cape May | 265.8 | 746 | -1.031981454 | Migrating |
| 2009 | Cape May | 281.2 | 265.8 | 0.056322014 | Migrating |
| 2010 | Cape May | 1026.5 | 281.2 | 1.294844077 | Migrating |
| 2011 | Cape May | 681.73 | 1026.5 | -0.409276551 | Migrating |
| 2012 | Cape May | 1222.26 | 681.73 | 0.583823198 | Migrating |
| 2013 | Cape May | 112.73 | 1222.26 | -2.383461304 | Migrating |
| 2014 | Cape May | 393.9 | 112.73 | 1.251101491 | Migrating |
| 1997 | Peninsula Point | 254.429 | 104.411 | 0.890686785 | Migrating |
| 1998 | Peninsula Point | 63.514 | 254.429 | -1.387761465 | Migrating |
| 1999 | Peninsula Point | 287.665 | 63.514 | 1.510536255 | Migrating |
| 2000 | Peninsula Point | 259.48 | 287.665 | -0.10311698 | Migrating |
| 2001 | Peninsula Point | 421.751 | 259.48 | 0.485735464 | Migrating |
| 2002 | Peninsula Point | 317.842 | 421.751 | -0.282860689 | Migrating |
| 2003 | Peninsula Point | 466.94 | 317.842 | 0.384646366 | Migrating |
| 2004 | Peninsula Point | 92.053 | 466.94 | -1.623836272 | Migrating |
| 2005 | Peninsula Point | 401.245 | 92.053 | 1.472207715 | Migrating |
| 2006 | Peninsula Point | 56.64 | 401.245 | -1.957856764 | Migrating |
| 2007 | Peninsula Point | 129.424 | 56.64 | 0.826378387 | Migrating |
| 2008 | Peninsula Point | 320.048 | 129.424 | 0.905377148 | Migrating |
| 2009 | Peninsula Point | 177.383 | 320.048 | -0.590159748 | Migrating |
| 2010 | Peninsula Point | 624.553 | 177.383 | 1.258724957 | Migrating |
| 2011 | Peninsula Point | 108.428 | 624.553 | -1.750949836 | Migrating |
| 2012 | Peninsula Point | 121.686 | 108.428 | 0.115357598 | Migrating |
| 2013 | Peninsula Point | 42.462 | 121.686 | -1.052834398 | Migrating |
| 2014 | Peninsula Point | 652.844 | 42.462 | 2.732728645 | Migrating |
| 2004 | South | 28.25 | 110.833 | -1.366931106 | Migrating |
| 2005 | South | 56.734 | 28.25 | 0.697280221 | Migrating |
| 2006 | South | 133.614 | 56.734 | 0.856581368 | Migrating |
| 2007 | South | 64.362 | 133.614 | -0.730431649 | Migrating |
| 2008 | South | 24.262 | 64.362 | -0.975612056 | Migrating |
| 2009 | South | 183.774 | 24.262 | 2.024795401 | Migrating |
| 2010 | South | 58.829 | 183.774 | -1.139071811 | Migrating |
| 2011 | South | 171.66 | 58.829 | 1.070880846 | Migrating |
| 2012 | South | 62.798 | 171.66 | -1.00559255 | Migrating |
| 2013 | South | 37.39 | 62.798 | -0.518519937 | Migrating |
| 2014 | South | 53.21 | 37.39 | 0.35284306 | Migrating |

**Table S3** Time-series for the egg density per host plant in North America[3]. $Egg_t$ is egg density per stem in year t. $R_{EGG}=\ln(Egg_t/Egg_{t-1})$ is the inter-annual variation in egg density. Each time-series refers to one region and roughly to one generation.

| ***Year*** | $Egg_t$ | $Egg_{t-1}$ | ***Site*** | $R_{EGG}$ | ***Season*** | ***Generation*** |
|---|---|---|---|---|---|---|
| 2003 | 1.159347 | 0.426293 | South | 1.000485 | Mar-Apr | 1 |
| 2004 | 0.097472 | 1.159347 | South | -2.47605 | Mar-Apr | 1 |
| 2005 | 0.712507 | 0.097472 | South | 1.989224 | Mar-Apr | 1 |
| 2006 | 0.180328 | 0.712507 | South | -1.37401 | Mar-Apr | 1 |
| 2007 | 1.679398 | 0.180328 | South | 2.231414 | Mar-Apr | 1 |
| 2008 | 0.434211 | 1.679398 | South | -1.35266 | Mar-Apr | 1 |
| 2009 | 1.727001 | 0.434211 | South | 1.380612 | Mar-Apr | 1 |
| 2010 | 0.669521 | 1.727001 | South | -0.94758 | Mar-Apr | 1 |
| 2011 | 0.308128 | 0.669521 | South | -0.77605 | Mar-Apr | 1 |
| 2012 | 0.714988 | 0.308128 | South | 0.841749 | Mar-Apr | 1 |
| 2013 | 0.13908 | 0.714988 | South | -1.63722 | Mar-Apr | 1 |
| 2014 | 0.150846 | 0.13908 | South | 0.081214 | Mar-Apr | 1 |
| 1998 | 0.063934 | 0.352596 | Midwest | -1.70748 | May-June | 2 |
| 1999 | 0.424424 | 0.063934 | Midwest | 1.892886 | May-June | 2 |
| 2000 | 0.185933 | 0.424424 | Midwest | -0.82535 | May-June | 2 |
| 2001 | 0.577283 | 0.185933 | Midwest | 1.132948 | May-June | 2 |
| 2002 | 0.38434 | 0.577283 | Midwest | -0.4068 | May-June | 2 |
| 2003 | 0.358267 | 0.38434 | Midwest | -0.07025 | May-June | 2 |
| 2004 | 0.185215 | 0.358267 | Midwest | -0.65976 | May-June | 2 |
| 2005 | 0.286415 | 0.185215 | Midwest | 0.435923 | May-June | 2 |
| 2006 | 0.510138 | 0.286415 | Midwest | 0.577239 | May-June | 2 |
| 2007 | 0.67841 | 0.510138 | Midwest | 0.285071 | May-June | 2 |
| 2008 | 0.271279 | 0.67841 | Midwest | -0.9166 | May-June | 2 |
| 2009 | 0.147665 | 0.271279 | Midwest | -0.6082 | May-June | 2 |
| 2010 | 0.342088 | 0.147665 | Midwest | 0.840122 | May-June | 2 |
| 2011 | 0.142315 | 0.342088 | Midwest | -0.87703 | May-June | 2 |
| 2012 | 1.022572 | 0.142315 | Midwest | 1.972037 | May-June | 2 |
| 2013 | 0.069053 | 1.022572 | Midwest | -2.6952 | May-June | 2 |
| 2014 | 0.114573 | 0.069053 | Midwest | 0.506335 | May-June | 2 |
| 1998 | 0.097185 | 0.308872 | Midwest | -1.15631 | Jul-Aug | 3 |
| 1999 | 0.26314 | 0.097185 | Midwest | 0.996066 | Jul-Aug | 3 |
| 2000 | 0.242717 | 0.26314 | Midwest | -0.08079 | Jul-Aug | 3 |
| 2001 | 0.427471 | 0.242717 | Midwest | 0.56599 | Jul-Aug | 3 |
| 2002 | 0.574263 | 0.427471 | Midwest | 0.295201 | Jul-Aug | 3 |
| 2003 | 0.280188 | 0.574263 | Midwest | -0.71763 | Jul-Aug | 3 |
| 2004 | 0.175283 | 0.280188 | Midwest | -0.46906 | Jul-Aug | 3 |
| 2005 | 0.279068 | 0.175283 | Midwest | 0.465054 | Jul-Aug | 3 |
| 2006 | 0.414484 | 0.279068 | Midwest | 0.39558 | Jul-Aug | 3 |
| 2007 | 0.393167 | 0.414484 | Midwest | -0.0528 | Jul-Aug | 3 |
| 2008 | 0.366823 | 0.393167 | Midwest | -0.06935 | Jul-Aug | 3 |
| 2009 | 0.263675 | 0.366823 | Midwest | -0.33016 | Jul-Aug | 3 |
| 2010 | 0.483431 | 0.263675 | Midwest | 0.606193 | Jul-Aug | 3 |
| 2011 | 0.34013 | 0.483431 | Midwest | -0.35158 | Jul-Aug | 3 |
| 2012 | 0.233927 | 0.34013 | Midwest | -0.37432 | Jul-Aug | 3 |
| 2013 | 0.084583 | 0.233927 | Midwest | -1.01727 | Jul-Aug | 3 |
| 2014 | 0.215759 | 0.084583 | Midwest | 0.936427 | Jul-Aug | 3 |
| 2000 | 0.031559 | 0.058457 | Northeast | -0.61644 | Jul-Aug | 3 |
| 2003 | 0.096575 | 0.09264 | Northeast | 0.041604 | Jul-Aug | 3 |
| 2004 | 0.037521 | 0.096575 | Northeast | -0.94543 | Jul-Aug | 3 |
| 2005 | 0.081482 | 0.037521 | Northeast | 0.775487 | Jul-Aug | 3 |
| 2006 | 1.054745 | 0.081482 | Northeast | 2.560672 | Jul-Aug | 3 |
| 2007 | 0.390325 | 1.054745 | Northeast | -0.99407 | Jul-Aug | 3 |
| 2008 | 0.379035 | 0.390325 | Northeast | -0.02935 | Jul-Aug | 3 |
| 2009 | 0.192461 | 0.379035 | Northeast | -0.67774 | Jul-Aug | 3 |
| 2010 | 0.234343 | 0.192461 | Northeast | 0.196893 | Jul-Aug | 3 |
| 2011 | 0.203741 | 0.234343 | Northeast | -0.13994 | Jul-Aug | 3 |
| 2012 | 0.326507 | 0.203741 | Northeast | 0.471604 | Jul-Aug | 3 |

| | | | | | | |
|---|---|---|---|---|---|---|
| 2013 | 0.005679 | 0.326507 | Northeast | -4.05167 | Jul-Aug | 3 |
| 2014 | 0.027574 | 0.005679 | Northeast | 1.580092 | Jul-Aug | 3 |
| 1999 | 0.017182 | 0.050505 | South | -1.0782 | Sep-Oct | 4(5) |
| 2000 | 1.879297 | 0.017182 | South | 4.694783 | Sep-Oct | 4(5) |
| 2001 | 0.181818 | 1.879297 | South | -2.33565 | Sep-Oct | 4(5) |
| 2002 | 0.207238 | 0.181818 | South | 0.130859 | Sep-Oct | 4(5) |
| 2003 | 0.607869 | 0.207238 | South | 1.076093 | Sep-Oct | 4(5) |
| 2004 | 0.878436 | 0.607869 | South | 0.368183 | Sep-Oct | 4(5) |
| 2005 | 0.65625 | 0.878436 | South | -0.2916 | Sep-Oct | 4(5) |
| 2006 | 0.465608 | 0.65625 | South | -0.3432 | Sep-Oct | 4(5) |
| 2007 | 0.185185 | 0.465608 | South | -0.92199 | Sep-Oct | 4(5) |
| 2008 | 0.158945 | 0.185185 | South | -0.1528 | Sep-Oct | 4(5) |
| 2009 | 1.325263 | 0.158945 | South | 2.12081 | Sep-Oct | 4(5) |
| 2010 | 0.544445 | 1.325263 | South | -0.8896 | Sep-Oct | 4(5) |
| 2011 | 0.353333 | 0.544445 | South | -0.43236 | Sep-Oct | 4(5) |
| 2012 | 0.34055 | 0.353333 | South | -0.03685 | Sep-Oct | 4(5) |
| 2013 | 0.527763 | 0.34055 | South | 0.438083 | Sep-Oct | 4(5) |
| 2014 | 0.252565 | 0.527763 | South | -0.73698 | Sep-Oct | 4(5) |

**Table S4** Best candidate linear models (lm) explaining growth rate of the overwintering population in Mexico including population size in the previous year ($N_{t-1}$), and *Time* (year) as predictors. We removed the two years for which the growth rate estimate was affected by severe winter storms in Mexico (2002 and 2004) and the two years with possible mass mortality events (2010 and 2016). Models are ranked according to their second-order Akaike's information criterion (AICc). Only models with AICc<7 are shown. Log-likelihood (logLik) and model weights are also reported.

| **Intercept** | $N_{t-1}$ | ***Time*** | $N_{t-1}$ **x** ***Time*** | **df** | **logLik** | **ΔAICc** | **Weight** |
|---|---|---|---|---|---|---|---|
| 0.03476 | -0.6065 | -0.4379 | | 4 | -16.793 | 0 | 0.436 |
| 0.03476 | -0.308 | | | 3 | -19.164 | 1.48 | 0.208 |
| -0.1391 | -0.8255 | -0.6032 | -0.2691 | 5 | -15.842 | 1.86 | 0.172 |
| 0.03476 | | | | 2 | -20.929 | 2.17 | 0.148 |
| 0.03476 | | -0.02438 | | 3 | -20.919 | 5 | 0.036 |

*df* indicated the number of estimated parameters calculated as the number of fixed effect coefficients + number of variance parameters.

**Table S5** Best candidate linear mixed-effects models (lme) explaining growth rate of the summer population in North America using population density in the previous year ($Density_{t-1}$), and *Time* as predictors. *Site* was included as random factor. We removed the two years for which the growth rate estimate was affected by severe winter storms in Mexico (2002 and 2004) and the two years with possible mass mortality events (2010 and 2016). Models are ranked according to their second-order Akaike's information criterion (AICc). Only models with AICc<7 are shown. Log-likelihood (logLik) and model weights are also reported.

| Intercept | $Density_{t-1}$ | *Time* | $Density_{t-1}$ x *Time* | df | logLik | ΔAICc | Weight |
|---|---|---|---|---|---|---|---|
| 0.1012 | -0.7385 | | | 4 | -95.02 | 0 | 0.534 |
| 0.1012 | -0.7400 | -0.0867 | | 5 | -94.42 | 1.05 | 0.316 |
| 0.1025 | -0.7603 | -0.0736 | 0.0759 | 6 | -94.00 | 2.53 | 0.151 |

*df* indicated the number of estimated parameters calculated as the number of fixed effect coefficients + number of variance parameters.

*Site* is a categorical factor with five levels (South, Midwest, Northeast, Cape May and Peninsula Point).

**Table S6** Best candidate linear mixed-effect models (lme) explaining inter-annual variation in egg density in relation to egg density in the previous year ($Egg_{t-1}$) and *Time* (year). *Site* was included as random factor. We removed the two years for which the growth rate estimate was affected by severe winter storms in Mexico (2002 and 2004) and the years with possible mass mortality events (only 2010 for egg time series). Models are ranked according to their second-order Akaike's information criterion (AICc). Only models with AICc<7 are shown. Log-likelihood (logLik) and model weights are also reported.

| **Intercept** | $Egg_{t-1}$ | ***Time*** | $Egg_{-1}$ **x** ***Time*** | **df** | **logLik** | **ΔAICc** | **Weight** |
|---|---|---|---|---|---|---|---|
| -0.0439 | -0.9143 | | | 4 | -90.489 | 0 | 0.672 |
| -0.0394 | -0.9028 | -0.0804 | | 5 | -90.304 | 2.00 | 0.247 |
| -0.0427 | -0.9015 | -0.0617 | 0.0544 | 6 | -90.193 | 4.23 | 0.081 |

*df* indicated the number of estimated parameters calculated as the number of fixed effect coefficients + number of variance parameters.

# References


1. Vidal, O. & Rendón-Salinas, E. Dynamics and trends of overwintering colonies of the monarch butterfly in Mexico. *Biol. Conserv.* **180,** 165–175 (2014).

2. Inamine, H., Ellner, S. P., Springer, J. P. & Agrawal, A. A. Linking the continental migratory cycle of the monarch butterfly to understand its population decline. *Oikos* **125,** 1081–1091 (2016).

3. Stenoien, C., Nail, K. R. & Oberhauser, K. S. Habitat productivity and temporal patterns of monarch butterfly egg densities in the Eastern United States. *Ann. Entomol. Soc. Am.* **108,** 670–679 (2015).
